# Supplementary material for: Topology-driven protein-protein interaction network analysis detects genetic sub-networks regulating reproductive capacity
Source: eLife. 2020 Sep 9;9:e54082. doi: 10.7554/eLife.54082 (PMC7550192; doi:10.7554/eLife.54082)
Supplement: Figure 4—source data 1. — AUC values for the ROC curves for each centrality measure for the three screens (Figure 4a). AUC values range from 0 to 1. A score above 0.5 indicates a positive correlation between the continuous variable (centrality) and the binary variable (above or below the Z score threshold). A score of 0.5 or less indicates no correlation between the variables. [file elife-54082-fig4-data1.docx]

| **Centrality metric** | ***hpo[RNAi]* Ovariole Number** | ***hpo[RNAi]* Egg Laying** | **Egg Laying** |
| --- | --- | --- | --- |
| Betweenness | 0.603 | 0.57 | 0.586 |
| EigenVector | 0.632 | 0.573 | 0.586 |
| Closeness | 0.612 | 0.551 | 0.588 |
| Degrees | 0.615 | 0.592 | 0.599 |
