# Supplementary material for: Topology-driven protein-protein interaction network analysis detects genetic sub-networks regulating reproductive capacity
Source: eLife. 2020 Sep 9;9:e54082. doi: 10.7554/eLife.54082 (PMC7550192; doi:10.7554/eLife.54082)
Supplement: Figure 7—source data 1. — Two genes that were above |Zgene| threshold (Table 2 and Figure 7—source data 1) in the hpo[RNAi] Egg Laying (CG12147) and hpo[RNAi] Ovariole Number seed list (CG6104) were not found in the PIN, and therefore not included in the network analysis or in this table (see Materials and methods for details). The removal of these two genes accounts for the difference between the number of positive candidates in Table 2 and Figure 7—source data 2, and the number of seed genes in these two sub-networks (Supplementary file 1 and Figure 7—source data 1). The proportion of connectors whose loss of function produced a significant phenotype (|Zgene| above threshold) is in parentheses and plotted in Figure 7a and b. All connectors except eukaryotic translation initiation factor three subunit j (eIF3J) in the hpo[RNAi] Egg Laying sub-network, for which no RNAi line was available at the time of testing, were tested. Therefore, the percentages of connectors above the threshold for the hpo[RNAi] Egg Laying sub-network were calculated out of 17 connectors. [file elife-54082-fig7-data1.docx]

| **Sub-Network** | **Number of Seeds** | **Number of Connectors** | **Number of connector genes above \|*Z_gene_*\| threshold within sub-network phenotype** |
| --- | --- | --- | --- |
| ***hpo[RNAi]* Egg Laying** | 58 | 18 | 7 (41.1%) |
| **Core** | 27 | 10 | 1 (10.0%) |
| **Egg Laying** | 49 | 11 | 0 (0.0%) |
| ***hpo[RNAi]* Ovariole Number** | 66 | 11 | 3 (27.2%) |
