# Supplementary material for: Topology-driven protein-protein interaction network analysis detects genetic sub-networks regulating reproductive capacity
Source: eLife. 2020 Sep 9;9:e54082. doi: 10.7554/eLife.54082 (PMC7550192; doi:10.7554/eLife.54082)
Supplement: Figure 7—source data 2. — Table indicates the number of unique genes among the connector genes and signalling genes screened, that had available RNAi lines at the time of analysis. The number of genes in the hpo[RNAi] Egg Laying sub-network that were above the primary filter of |Zgene| > 1 are also indicated. Percentage of the number of connectors and signalling candidates above threshold for each phenotype from the number of connectors above the primary filter is in parentheses and plotted in Figure 7c. All connectors except eukaryotic translation initiation factor three subunit j (eIF3J) in the hpo[RNAi] Egg Laying sub-network, for which no RNAi line was available at the time of testing, were tested. Therefore, the percentages of connectors above the threshold were calculated out of 32 unique connectors. [file elife-54082-fig7-data2.docx]

| **Unique Genes with RNAis** | | **Number of genes above threshold in** | | | | |
| --- | --- | --- | --- | --- | --- | --- |
|  |  | ***hpo[RNAi]* Egg Laying *Z_gene_*>\|1\|** | **Egg Laying *Z_gene_*>\|5\|** | ***hpo[RNAi]* Egg Laying *Z_gene_*>\|5\|** | ***hpo[RNAi]* Ovariole Number *Z_gene_*>\|2\|** | **All three screens** |
| **Connectors** | 42 | 32 | 10/32 (31.2%) | 13/42 (30.9%) | 12/32 (37.5%) | 8/32 (25%) |
| **Signalling candidates** | 463 | 273 | 49/273 (17.9%) | 59/463 (12.7%) | 67/273 (24.5%) | 27/273 (9.8%) |
